# Micromotors with asymmetric shape that efficiently convert light into work by thermocapillary effects

**DOI:** 10.1038/ncomms8855

**Published:** 2015-07-29

**Authors:** Claudio Maggi, Filippo Saglimbeni, Michele Dipalo, Francesco De Angelis, Roberto Di Leonardo

**Affiliations:** 1 Dipartimento di Fisica, Università di Roma ‘Sapienza', I-00185 Roma, Italy; 2 Istituto Italiano di Tecnologia, I-16163 Genova, Italy; 3 NANOTEC-CNR, Institute of Nanotechnology, Soft and Living Matter Laboratory, Piazzale A. Moro 2, I-00185 Roma, Italy

## Abstract

The direct conversion of light into work allows the driving of micron-sized motors in a contactless, controllable and continuous way. Light-to-work conversion can involve either direct transfer of optical momentum or indirect opto-thermal effects. Both strategies have been implemented using different coupling mechanisms. However, the resulting efficiencies are always very low, and high power densities, generally obtained by focused laser beams, are required. Here we show that microfabricated gears, sitting on a liquid–air interface, can efficiently convert absorbed light into rotational motion through a thermocapillary effect. We demonstrate rotation rates up to 300 r.p.m. under wide-field illumination with incoherent light. Our analysis shows that thermocapillary propulsion is one of the strongest mechanisms for light actuation at the micron- and nanoscale.

## Abstract (web summary)

The direct conversion of light into work allows the control of micromotors, but typically with low efficiencies and high power density requirements. Here, Maggi *et al*. demonstrate efficient thermocapillary propulsion of microgears on a liquid–air interface with wide-field, incoherent illumination.

## Graphical abstract

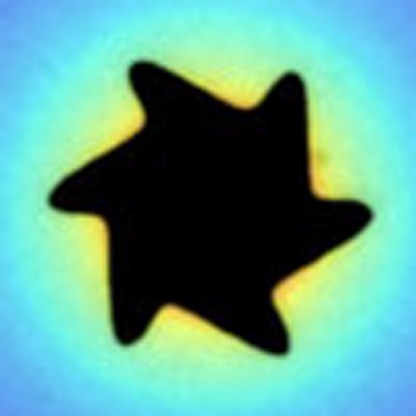

Recent years have seen an explosion of interest in designing strategies to produce self-propelled micro- and nanomachines^1^,^2^,^3^. These are micron- and nano-sized objects that are capable of harnessing some form of environmental energy and convert it into mechanical work. Self-propelled microorganisms provided an inspiration for building micro- and nanoswimmers actuated by external magnetic fields^4^,^5^,^6^. A different approach focuses on the fabrication of hybrid devices integrating microfabricated units with biological motors^7^,^8^,^9^,^10^,^11^,^12^,^13^,^14^. On the other hand, many examples of self-phoretic particles were demonstrated providing a fully synthetic route to self-propulsion. These are typically polar (Janus) structures, having an inhomogeneous catalyst distribution, that are capable of producing and carrying along a local concentration gradient that drives phoretic motion^15^,^16^,^17^. Alternatively catalysts can be integrated into the inner wall of micro/nanotubes and generate propulsion by recoiling oxygen gas bubbles^18^. A typical drawback of catalytic propulsion consists in fuel consumption leading to a progressive slow down of activity. In this respect, self-propelled particles that absorb energy from an external optical field have the advantage of being long lived and easily switchable. Many different mechanisms for the conversion of light into work have been proposed. Optical trapping relies on the direct momentum transfer from radiation to matter^19^. The momentum flow of a wave carrying a power *P* is in general *P*/*v*. In the case of light, *v*=*c* leading to a very inefficient conversion of optical power into mechanical work and the consequent need of focused laser beams. A more efficient strategy for light-to-work conversion is provided by opto-thermal effects. Janus beads, half covered in gold, were shown to generate self-propulsion due to asymmetric heating under illumination with consequent thermophoresis in the locally generated temperature gradient^20^. Asymmetric heating can also generate propulsion via the local demixing of a critical binary mixture^21^. Another interesting strategy is that of using photocatalytic materials to fabricate Janus colloids displaying self-phoretic motion under illumination^22^. This last approach, however, relies again on the presence of a chemical fuel, hydrogen peroxide, that is progressively consumed. One of the strongest forces acting at the micron scale is surface tension. A floating object, intersecting a liquid–air interface is pulled along the contact line by a capillary force measuring typically tens of nanonewton per micron^22^,^23^. If the surface tension is uniform, the total force and torque are identically zero regardless of the object's shape. Surface tension, however, is very sensitive to temperature and chemical composition so that a small temperature or concentration gradient around a floating object can have strong mechanical effects^24^. These effects are generally referred to as Marangoni propulsion and have been demonstrated to provide an efficient mechanism for light-to-work conversion in macroscopic objects under non-uniform heating with light^25^. On the micron scale, localized heating produced by a focused laser beam can give rise to thermocapilary forces that are capable of moving and routing droplets in microfluidic devices^26^,^27^. In all these approaches, however, a local heating is applied around a target droplet, requiring the use of a microscope for focusing coherent light onto a target region.

Here we show that Marangoni forces can be efficiently used to propel micron-sized motors in a continuous way using an incoherent wide-field illumination. We fabricated microstructures with a homogeneous light absorbing coating that self-generate the required temperature gradient in the surrounding fluid. Continuous propulsion is obtained using a shape that breaks mirror symmetry so that, when the structure floats at a liquid–air interface, a net torque arises due to surface tension gradients. We demonstrate rotation rates up to 300 r.p.m., which can be quickly, and indefinitely controlled by tuning the incident light power.

## Results

### Observation of light-induced rotation

**Figure 1:**
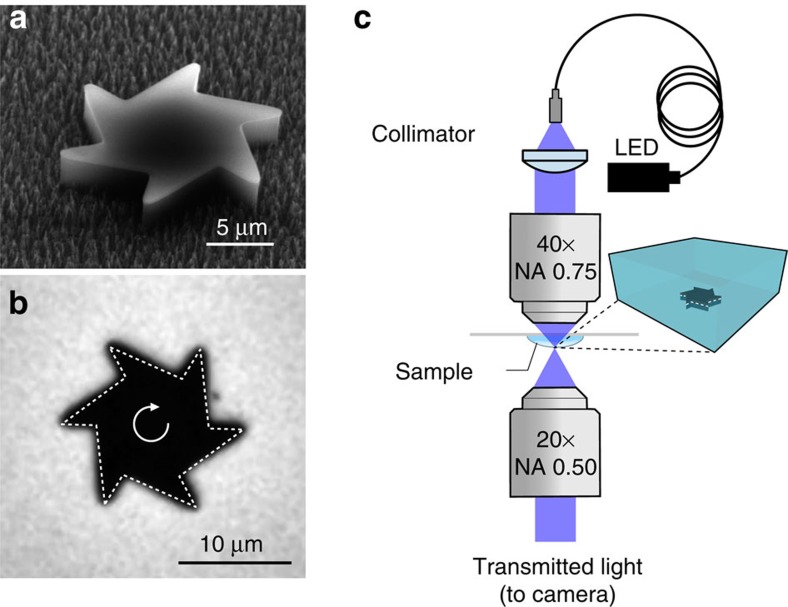
Microgears and experimental set-up. (**a**) Scanning electron microscope image of a microfabricated gear. (**b**) Bright-field microscopy image of the microgear lying at the liquid–air interface, the dashed line shows tracking by a shape-recognition algorithm and the arrow represents the observed rotation direction. (**c**) Experimental set-up. A 20 × objective is used for imaging, while a 40 × objective condenses blue light from a high power LED. A drop of sample hangs from a glass coverslip so that microgears sediment at the air–liquid interface.

**Figure 2:**
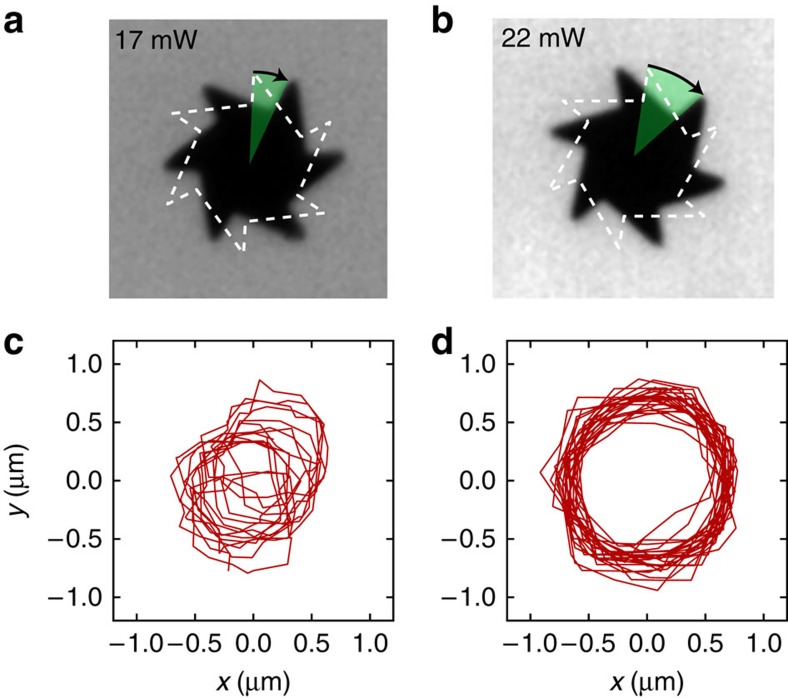
Rotational and translational dynamics. Top row shows the rotational displacement in a time interval of 20 ms for the same gear at total incident powers of 17 mW (**a**) and 22 mW (**b**). The corresponding trajectories of the gears centres are shown in the bottom row for a total time interval of 5 s (**c**,**d**).

**Figure 3:**
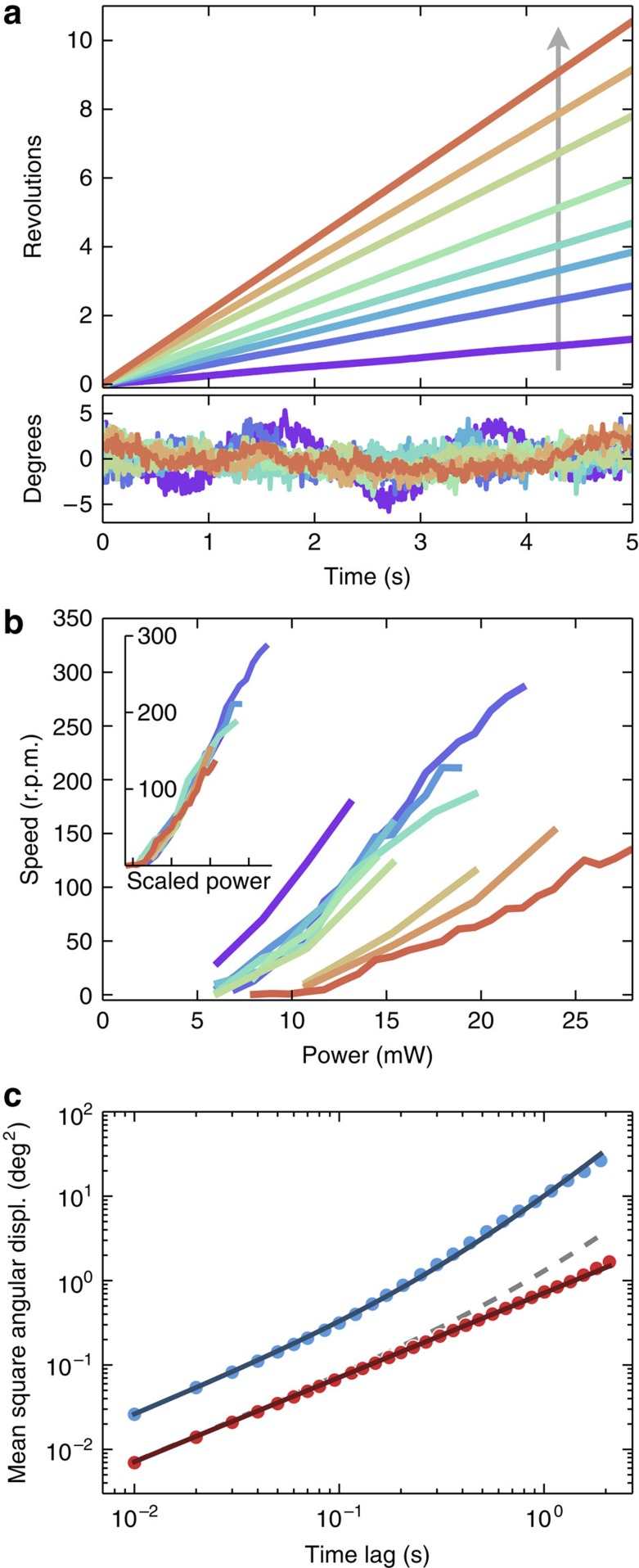
Measurements of rotational speed and diffusivity. (**a**) Angular displacement as a function of time for a single gear under different illumination power levels. The arrow points towards increasing illumination powers. The bottom panel displays the corresponding angular deviations from a linear fit. (**b**) Angular speed as a function of the illumination power for ten different gears. All curves scale on top of each other when rescaling power by an arbitrary factor accounting for absorption variations (inset). (**c**) Mean squared angular displacement for a gear at the liquid–air interface is shown in blue together with a best fit curve in equation (1). Red data refer to a gear at the liquid–solid interface. The dashed line represents the expected behaviour assuming that a comparable light-induced torque was present in the bulk.

Using laser lithography, we fabricate asymmetric microgears having an outer radius *a*=8 μm and a thickness of 2.6 μm (Fig. 1a). The gears are coated on one side with a uniform layer of amorphous carbon to increase light absorption. The structures are then detached from the silicon substrate by washing in a photoresist remover solvent (see Methods). A small drop of solvent containing free floating gears is then deposited on a microscope glass slide and turned upside down so that microgears sediment on the free solvent–air interface. A high power light-emitting diode (LED) lamp projects a wide-field illumination disk with a radius *A* ∼140 μm and a maximum total power of 30 mW (before sample). The fraction of total power that illuminates a single gear is given by ∼(*a*/*A*)^2^∼3 × 10^−3^. Gears lying within the illumination disk start to rotate steadily around their centres for total incident powers as low as few mW (∼15 μW on a single gear; see Supplementary Video 1). The structures can sediment with two possible orientations (carbon face up or down) but they always rotate with the long teeth edges forward, as shown in Fig. 1b (see also Supplementary Video 2). Using digital video microscopy, we record movies of rotating gears at a 100 Hz frame rate (Fig. 1c). We subsequently process digital frames with a shape-recognition algorithm to extract the trajectories of the gears under varying illumination power (see Fig. 2 and Supplementary Video 1). All gears rotate with a well defined speed displaying angular fluctuations that depart from a linear fit by less than 5° (Fig. 3a). The angular speed increases approximately linearly with illumination power (Fig. 3b). Due to inhomogeneities in the carbon coatings, a small linear velocity is superimposed to the rotation. This translational velocity is constant in modulus but rotates rigidly with the structure. Consequently, the centre of mass traces a small circle with a typical radius of 0.5 μm (Fig. 2c,d). Figure 3a reports the cumulative angular displacements of the same gear for different illumination powers. In Fig. 3b, we report the average angular speeds as a function of power for 10 different gears obtained from the same batch. All tracked gears display a qualitatively similar speed dependence on power. For about half of the tracked microgears, the rotation speed for a given illumination power is reproducible. The remaining microgears rotate with different speeds, which can be attributed to differences in the carbon coatings that result in different light absorption coefficients. This hypothesis is confirmed by the fact that all the curves in Fig. 3b collapse on top of each other when rescaling the power axis by a multiplicative factor (Fig. 3b, inset). The remaining small differences between the curves can be attributed to small differences in the gears shape resulting from imperfections in the fabrication process.

## Discussion

The maximum observed speed is Ω=30 rad s^−1^ (∼300 r.p.m.) for a corresponding incident power on the gear's surface of *P*_O_=60 μW. Calling Γ the rotational viscous drag acting on the gear, we can estimate the efficiency *ɛ* of the light-to-work conversion as the ratio between the optical power *P*_O_ and the mechanical power *P*_M_=ΓΩ^2^ that would be required to spin the gear at the same speed by an external torque. If we estimate Γ as the viscous drag on a spherical object of radius *a* sitting in the middle of a liquid–air interface (Γ=4*πηa*^3^=11 pN μm s rad^−1^), we get *ɛ*=*P*_O_/*P*_M_∼10^−10^. Combining reflection and transmission microscopy as in ref. 28 we measured typical absorption coefficients of 2% in our coatings (see Supplementary Fig. 3 and Supplementary Discussion). That means that it should be possible to increase the efficiency by about two orders of magnitude with a better coating. Even then, an efficiency of 10^−8^ may seem very small but it is not quite so small when compared with alternative mechanisms for light-to-work conversion. For example, light can be used to rotate absorbing or birefringent objects by direct transfer of optical spin angular momentum^29^. The flow of spin angular momentum in a circularly polarized beam of power *P*_O_ is given by *T*_O_=*P*_O_/*ω*_O_ with *ω*_O_ the optical angular frequency. The corresponding efficiency will be 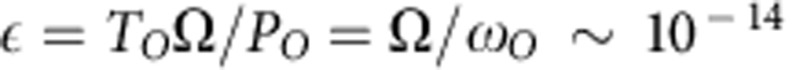 for comparable rotational speeds. Thermophoresis could be used to rotate similar gears in a bulk fluid via opto-thermal effects^20^,^30^. Similarly, we can estimate the efficiency of light-to-work conversion for thermophoresis as a ratio between angular frequencies *ɛ*=Ω/*ω*_T_ where *ω*_T_∼*κa*/*S*_T_*k*_B_*T* with *S*_T_ the Soret coefficient and *κ* the solvent's thermal conductivity. Substituting typical values for *κ* in non-conductive liquids and for *S*_T_^31^ we obtain an efficiency of ∼10^−13^ for rotation rates of the order of those observed in our experiments. On the basis of these arguments, we can conclude that thermophoresis is playing no role in our experiments since a 10^5^ larger power density would be required to observe rotations of the same magnitude, with obvious heating problems. Much higher efficiency values can be obtained by a Marangoni type propulsion mechanism. In that case, we can estimate by dimensional arguments an efficiency *ɛ*=Ω/*ω*_M_ with *ω*_M_∼*κ*/*γ*_T_*a* where *γ*_T_ is the derivative of surface tension with temperature. Substituting typical values for liquids we now get *ɛ* ∼10^−8^, which is of the same order of the expected maximum efficiency and makes Marangoni propulsion the best candidate to explain the origin of the observed rotations. Another strong evidence in favour of the capillary origin is that no rotations are observed in the bulk or when we let gears sediment on a solid glass substrate. One may argue that rotations at a solid interface may be hindered by the increased drag due to the no slip boundary condition imposed by the solid wall. For this reason, we directly compared the mean square angular displacements of gears sitting on a liquid and a solid interface under similar illumination levels. For a rotating Brownian gear, the mean square angular displacement will display a transition from a diffusive behaviour at short timescales to a ballistic regime at large time lags:


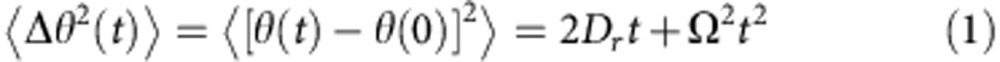


where *D*_r_=*k*_B_*T*/Γ is the rotational diffusion coefficient. Equation (1) fits very well the data for a liquid surface as shown in Fig. 3c giving Γ=11±1 pN μm s rad^−1^ that is consistent with our previous estimate. The ballistic component disappears at the solid interface where the only visible component is Brownian rotational diffusion with an increased fitted drag coefficient Γ=38±1 pN μm s rad^−1^. This observed fourfold increase in the drag should give rise to a fourfold reduction in the rotating speed, which should be still detectable in our experiments (see dashed line in Fig. 3c). Those observations confirm that any other mechanism that could be active in the bulk, that is, thermophoresis, cannot account for the large rotational speeds found in our experiments.

### Capillary torque on uniformly heated gears

**Figure 4:**
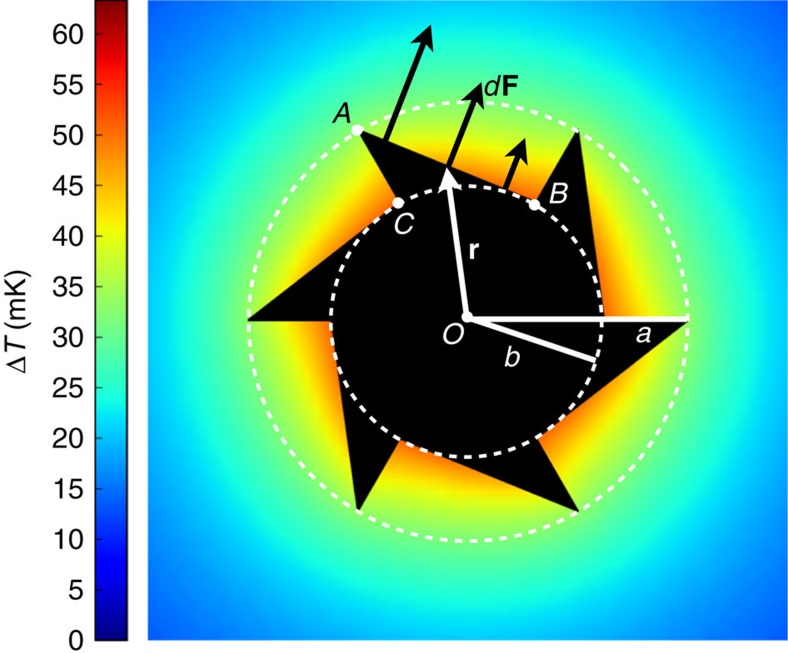
Origin of capillary torque on the gear. A uniformly heated gear produces a temperature gradient resulting in smaller surface tension forces over the inner portions of the gear's contour. The temperature profile, at the air–liquid interface, is computed by three-dimensional finite-element simulations and is represented by the colour level (see colour bar). Due to the asymmetric shape of the gear a non-zero total torque is generated (Equation (5)).

As a further argument in support of Marangoni propulsion, we now show that the expected torque applied by capillary forces to a static heated gear will give rise to rotations in the same direction as those observed in our experiments. We assume that a liquid–air–solid contact line forms along the saw-toothed contour of our gears. In absence of heating, the temperature is uniform and each element of the contact line will pull with the same force per unit length resulting in a zero net force and torque. In the presence of heating, a non-uniform temperature field will develop giving rise to surface tension gradients along the gear's contour. Indicating by **r** the position vector of a generic contact line element *ds*, the total torque exerted by capillary forces will be given by:


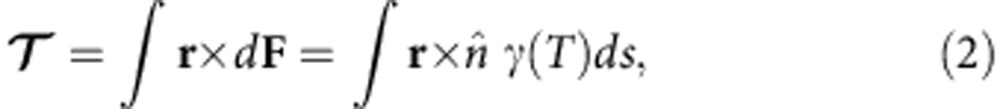


where 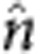 is outward pointing normal to the line element and *γ*(*T*) represents the surface tension of the liquid–air interface at the local temperature *T* in **r**. We can break the total torque into the separate contributes coming from each linear section of the contour and write 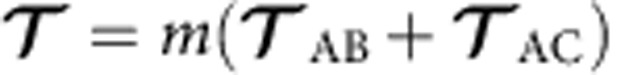 with *m* the number of gear's teeth (Fig. 4). We now observe that for symmetry reasons the points B and C will have the same temperature and hence surface tension. We then assume that temperature variations along the contour are smooth enough to be well approximated by linear profiles. Since the total torque is zero in the case of a homogeneous surface tension, we can replace the value *γ*(*T*) in equation (2) with *γ*(*T*)−*γ*(*T*_B_) and obtain for the partial contributions to the integral:


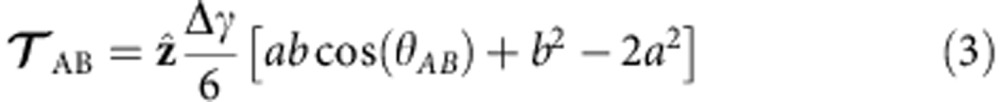



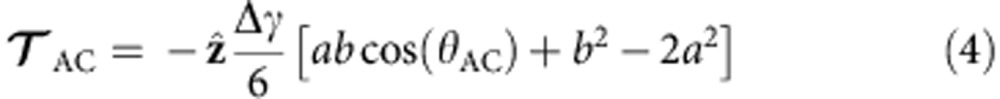


where 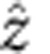 is the unit vector pointing out of the plane in Fig. 4 and Δ*γ*=*γ*(*T*_A_)−*γ*(*T*_B_) is the surface tension difference between outer (A) and inner (B and C) vertices. The symbols *θ*_AB_ and *θ*_AC_ represent, respectively, the angles ∠AOB and ∠AOC. In our specific case, we have six teeth with *θ*_AB_=*π*/3 and *θ*_AC_=0 resulting in a total torque:


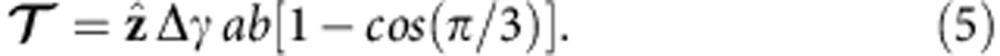


When the gear is uniformly heated, inner vertices will be hotter than outer ones. Since surface tension usually decreases with temperature, Δ*γ*<0 and the rotation proceeds with the long teeth edge forward (that is, clockwise in Fig. 4). Typical values for ∂*γ*/∂*T* are in the range of mN(mK)^−1^ so that for a gear with micron-sized radius we get a capillary torque of thousands of pN μm per every degree of temperature difference. A substantial torque can be generated even with temperature differences in the millikelvin range. We can estimate the temperature differences in our experiment as given by ∇*T* ∼*αP*_O_/*κa*^2^ which is of the order of 10 mK(μm)^−1^. This estimate is validated by direct three-dimensional finite-element calculations of the temperature field at the liquid–air interface as reported in Fig. 4 for a total absorbed power *αP*_O_=0.5 μW corresponding to an intermediate incident power of 10 mW. By simple dimensional reasoning, we expect the angular speed to scale, for fixed power density *I*, as 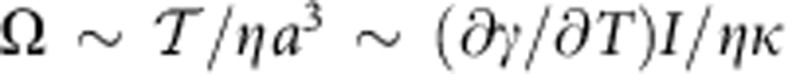. Since system size does not appear explicitly, we expect that, under similar illumination conditions, comparable rotation speeds could be achieved even for structures of much larger or smaller size.

## Conclusions

We have shown that asymmetric microrotors, uniformly coated with a light-absorbing material and sitting on a liquid–air interface, can smoothly rotate under wide-field illumination with incoherent light. We demonstrate rotation rates up to 300 r.p.m. that can be continuously controlled with the intensity of incident light. The propulsion mechanism originates from the strong capillary torque that arises even in the presence of temperature variations in the millikelvin range. The observed efficiency of the light-to-work conversion is about five orders of magnitude larger than previously reported effects allowing to generate indefinite rotations even at low power densities. The obtainable rotation rates should in principle remain constant by scaling down the system size opening the way to applications at the nanoscale.

## Methods

### Microfabrication

The fabrication starts with the spin-coating and baking (200 °C) of LOR3B/SU-8 (200 nm/4 μm on a silicon wafer). An amorphous carbon film (100 nm) is then deposited on the SU-8 by sputtering. Subsequently, LOR3B/S1813 (200 nm/1.5 μm) is spin-coated on the carbon layer, and laser lithography is performed to obtain the negative pattern of the microgears in the LOR3B/S1813 bilayer. After the evaporation of 100 nm chrome, the LOR3B/S1813 bilayer is removed by *N*-methyl-2-pyrrolidone, leaving chrome microgears on the carbon layer. The SU-8/carbon layer is then etched by reactive-ion etching in oxygen plasma; the chrome microgears act as etching mask and transfer their shape onto the SU-8; the etching process is anisotropic and produces SU-8 vertical walls. A finalized SU-8 microgear is depicted in Fig. 1a after chrome removal in chrome-etch solution. The microgears are finally released from the wafer by PG remover, which dissolves the sacrificial LOR3B layer at the bottom of the microgears (see Supplementary Figs 1 and 2 and Supplementary Methods for a detailed scheme of microfabrication).

### Optical set-up

Video-microscopy images are taken by a custom-built inverted optical microscope (see Fig. 1b,c), equipped with a 20 × (numerical aperture=0.25) objective, at a frame rate of 100 frames per second. The samples can be illuminated with variable light intensity provided by a fibre-coupled blue LED with a maximum output power of 200 mW. LED light is collimated by an aspheric lens and then refocused by a 40 × (numerical aperture=0.75) objective acting as a condenser. This results in a sharp illumination disk with a radius of about 140 μm and a maximum power of ∼30 mW before sample (see Supplementary Discussion and Supplementary Fig. 3 for a detailed description of the optical measurements of the absorption coefficient).

## Additional information

**How to cite this article:** Maggi, C. *et al*. Micromotors with asymmetric shape that efficiently convert light into work by thermocapillary effects. *Nat. Commun.* 6:7855 doi: 10.1038/ncomms8855 (2015).

## Supplementary Material

Supplementary Figures, Supplementary Discussion, Supplementary Methods and Supplementary Reference Supplementary Figures 1-3, Supplementary Discussion, Supplementary Methods and Supplementary Reference

Supplementary Movie 1 One microgear rotating at the air-liquid interface under varying incident illumination power.

Supplementary Movie 2 Two microgears, sedimented with opposite orientations at the air-liquid interface, rotating under similar illumination conditions.
