# Supplementary material for: Micromotors with asymmetric shape that efficiently convert light into work by thermocapillary effects
Source: Nat Commun. 2015 Jul 29;6:7855. doi: 10.1038/ncomms8855 (PMC4532854; doi:10.1038/ncomms8855)
Supplement: Supplementary Figures, Supplementary Discussion, Supplementary Methods and Supplementary Reference — Supplementary Figures 1-3, Supplementary Discussion, Supplementary Methods and Supplementary Reference [file ncomms8855-s1.pdf]

SUPPLEMENTARY FIGURES

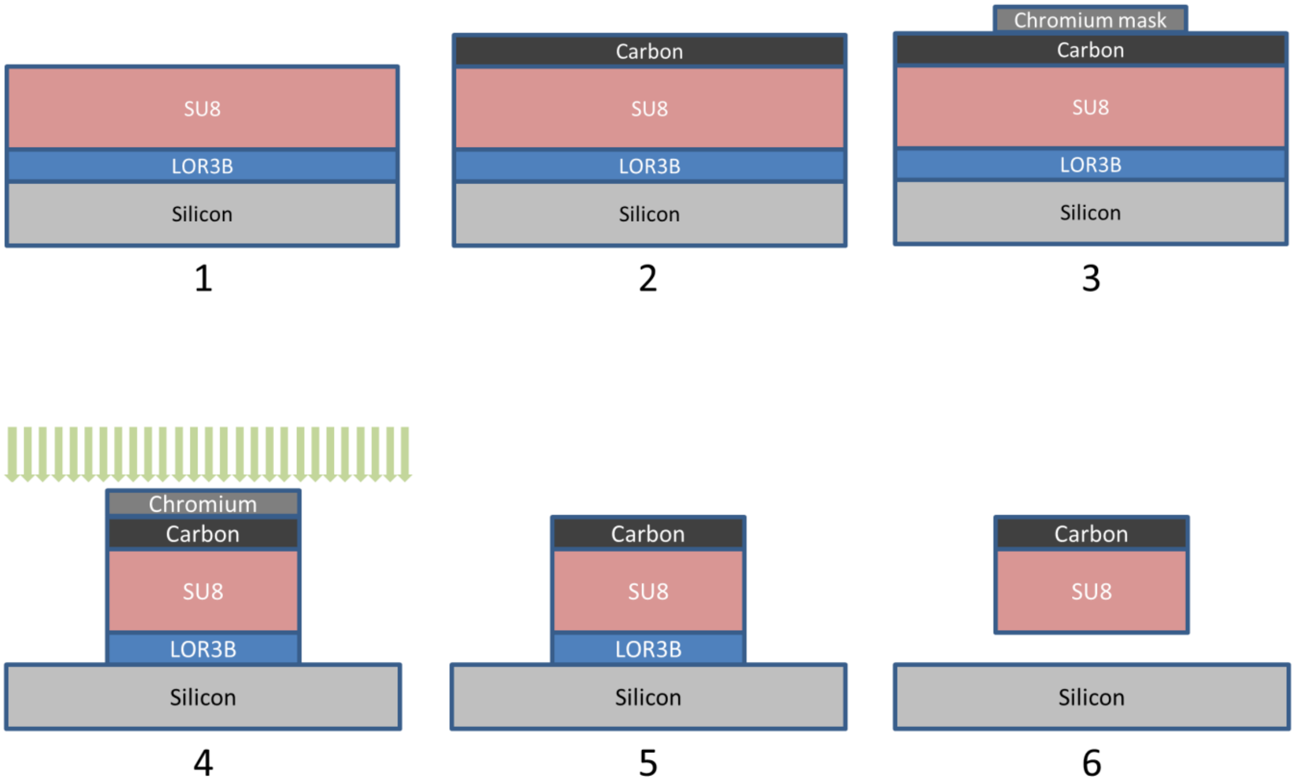

Supplementary Figure 1: Scheme of microfabrication: (1) Spin coating of LOR3B and SU8 on a silicon substrate. 2: Deposition of amorphous carbon. (3) Laser optical lithography and deposition of rotor-shaped chrome. (4) RIE etching of the layer stack. (5) Wet removal of chrome in chrome-etch solution. (6) Releasing of the micro-rotors with LOR3B solvent.

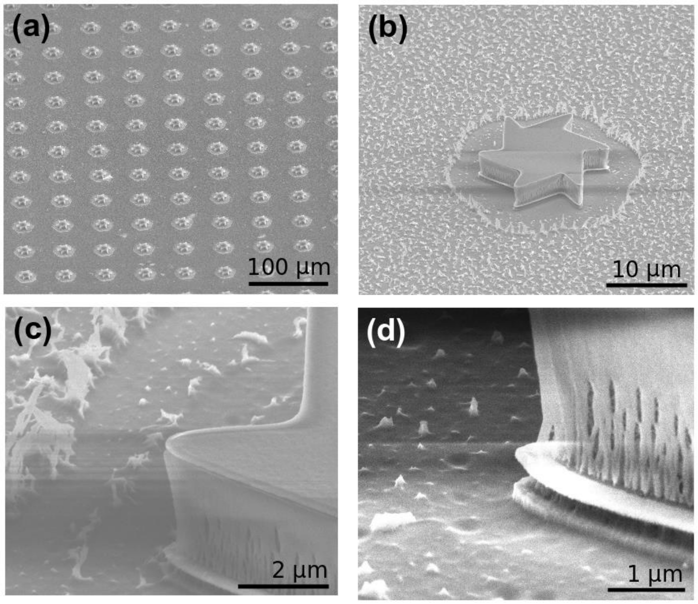

Supplementary Figure 2: (a) Bird's eye view of several micro-rotors fabricated on a silicon wafer. (b) Close view of an SU8 micro-rotor. The white flakes around the micro-rotor are residues from the RIE etching process. (c) Zoomed view on one micro-rotor tooth. (d) Detailed view of the micro-rotor base. The LOR3B sacrificial layer is clearly visible at the bottom of the microrotor (bright layer). It is possible to observe that under the LOR3B the silicon wafer has been also slightly etched during the RIE process.

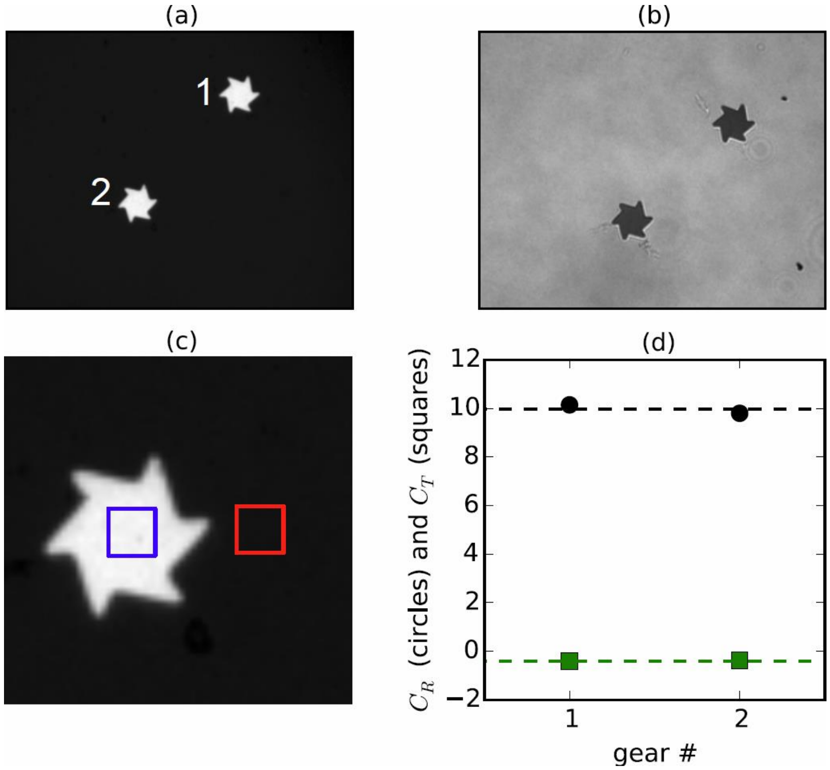

Supplementary Figure 3: (a) Microgears at the liquid-air interface imaged by reflection optical microscopy. (b) The same microgears of (a) at the liquid-air interface imaged by transmission optical microscopy. (c) Zoom on the gear no. 1, seen in reflection microscopy, the squares indicate the regions considered to measure IG (the intensity of the light reflected by the gear) and I_0 (the intensity of the light reflected by the liquid). (d) Reflection and transmission contrast (Eq. (1) and (2)) measured for the three gears in (a) and (b), dashed lines represent the average value.

SUPPLEMENTARY DISCUSSION

Measurement of the absorption coefficient of the microgears

To estimate the absorption coefficient of the graphite coated microgears we follow the approach of Ref. [1]. We image the microgears suspended at the air-liquid interface with reflection and transmission optical microscopy as shown in Supplementary Figure 3(a) and (b) respectively. Consider now the images taken by reflection microscopy. From these we can isolate portions of the image located at the center of the each single gear and in a region nearby the gear as shown in Supplementary Figure 3(c). The average light intensity registered by the camera in these regions is proportional to the light reflected by the gear I_G and by the liquid I_0 respectively. This allows to estimate the *reflection contrast*:

$$C_R = \frac{I_G - I_0}{I_0} = \frac{R_G - R_0}{R_0} \qquad \text{Eq. 1}$$

that is related to the reflectance of the gear (R_G) and the liquid (R_0) interfaces. Similarly the *transmission contrast* can be estimated by considering the same portions of the image obtained by transmission microscopy

$$C_T = \frac{I_G - I_0}{I_0} = \frac{T_G - T_0}{T_0} \qquad \text{Eq. 2}$$

that is related to the transmittance of the gear (T_G) and the liquid (T_0). Supplementary Figure 3(d) reports the values of C_R and C_T obtained by Eq. (1) and (2) from the images of the two gears seen in Supplementary Figure 3(a) and (b).

To obtain the absorbance of the gear A_G let us consider that the total light intensity is conserved when considering absorption, transmission and reflection from the gear-air interface:

$$A_G + T_G + R_G = 1 \qquad \text{Eq. 3}$$

The same holds for the liquid-air interface where the absorption is negligible:

$$T_0 + R_0 = 1 \qquad \text{Eq. 4}$$

Moreover the reflectance of the air-liquid interface can be estimated from Fresnel equations:

$$R_0 = \left(\frac{n_l - n_a}{n_l + n_a}\right)^2 \quad \text{Eq. 5}$$

where n_a = 1.00 is the refractive index of air and n_l =1.47 is the refractive index of the liquid (N-Methyl-2-pyrrolidone). Combining these formulas we can obtain the absorbance of the gear as:

$$A_G = (R_0 - 1)C_T - R_0 C_R \quad \text{Eq. 6}$$

Using the values of C_T and C_R shown in Supplementary Figure 3(d) we get for the two gears in the image $A_G = 3.6 \times 10^{-2}$ and $A_G = 1.6 \times 10^{-2}$.

SUPPLEMENTARY METHODS

Details of microfabrication

The fabrication process (scheme shown in Supplementary Figure 1) starts with the spin coating of 200 nm LOR3B and 4 μm SU-8 on a silicon wafer. The SU-8 layer is hard baked at 200◦C for 30 min in order to make it chemically stable; the hard-baking also makes the SU-8 thinner down to about 2.6 μm. A thin amorphous carbon film (100 nm) is then deposited homogeneously on the SU-8 with sputtering methods. Subsequently 200 nm LOR3B and 1.5 μm S1813 are spin coated on the amorphous carbon layer, and laser optical lithography is performed to obtain the negative pattern of the microgears in the LOR3B/S1813 bilayer. After the deposition of 100 nm chrome by e-beam evaporation, the LOR3B/S1813 bilayer is removed by N-methyl-2-pyrrolidone, leaving a matrix of 40 × 40 thin chrome microgears on the amorphous carbon layer. The SU-8/carbon layer is then dry etched with a Reactive Ion Etching (RIE) process performed in an ICP system in oxygen atmosphere; the chrome microgears act as etching mask and transfer their shape onto the SU-8. The etching process is strongly anisotropic and produces vertical walls in the SU-8 microgears. The wafer with the SU-8/carbon microgears is then immersed in chrome-etch solution to remove the chrome mask. The result of this fabrication process is shown in Supplementary Figure 2(a)-(d), that depicts an SU-8 microgear after RIE etching and chrome removal. The microgears are then released from the silicon wafers by rinsing and sonicating the whole

sample in N-methyl-2-pyrrolidone, which dissolves the sacrificial LOR3B layer at the bottom of the microgears.

SUPPLEMENTARY REFERENCE

[1] H. S. Skulason, P. E. Gaskell and T. Szkopek, Nanotechnology, **21** 295709 (2010)
